# Postgraduate medical education selection in Canada: Opening the black box

**DOI:** 10.36834/cmej.70304

**Published:** 2020-07-15

**Authors:** Kulamakan Kulasegaram, Marcel D’Eon

**Affiliations:** 1 Department of Family & Community Medicine, Faculty of Medicine University of Toronto, Ontario, Canada; 2 The Wilson Centre, University Health Network, Ontario, Canada; 3 University of Saskatchewan, Saskatchewan, Canada; 4 Medical College of Georgia, Augusta University, Georgia, USA

“Anxious.” This was the most frequent word I heard at a recent roundtable with some undergraduate medical students on their upcoming applications to postgraduate (PG) medical training. This is not news to the Canadian medical education community. The annual “match” has become a life changing event for medical students, their families, and their communities. A significant chunk of undergraduate (UG) medical education is devoted to ensuring students have the adequate clinical experiences for career exploration and development. The seriousness of failing to match to the first choice or worse – failing to match all - is viewed by most medical students as catastrophic.^1^ Beyond the potential delay or change to their career plans, many unmatched medical students feel shame and stigma.^2^ As the conversation at this roundtable expanded to include a discussion of the selection process, many students – and faculty – voiced a common concern: “the system is broken.” A larger proportion of candidates have gone unmatched to a PG program in recent years, a fact that has provoked major concerns from students and schools. As the ratio of graduates to available PG positions continues to decrease, the need for advocacy to change the system is growing.^3^^,^^4^ The participants at my roundtable were no different from students and faculty across Canada who are worried and anxious about the selection process. Anxiety provokes questions like “what are they looking for…what should I do, what electives or extracurricular activities should I have, how do they choose, what are my chances this year?” Answering these questions is difficult and maybe even impossible. Each discipline and PG program has unique aspects. But a larger challenge is that the answers are largely unknown or not publicized broadly. This dearth of information impacts PG programs as well. Students are paying much more attention to the selection processes to optimize their chances of getting selected and how this is accomplished. In the age of competency based education, tighter fiscal restraints, and more complex healthcare, applicants and the training programs are seeking answers but finding few.^5^

Ultimately, all of our answers depend on first understanding how the selection system functions in Canada. At the macro level, the selection processes involves multiple stakeholders including provincial ministries of health, medical students, medical schools, and postgraduate programs. The macro level issues have been the subject of intense debate and advocacy as all stakeholders work to find a more optimal working of the selection system. Provincial healthcare systems which largely fund PG training depend on residents as a significant part of their present and future healthcare workforce. The financial costs of training residents are an investment in physicians who may reside and work in the province. Considerations for future healthcare needs and financial capacity dictate how provinces fund postgraduate training seats. The misalignment between the nature and number of training opportunities by province and the personal career and lifestyle aspirations of medical students is perhaps a root cause for the selection crisis. Supporting unmatched students remains a challenge for many medical schools which invest heavily in preparing and supporting students. On the flip side, there is pressure on postgraduate programs including the national competition for the ‘best and the brightest’ and the desire to fill their allocated number of training spots. Intermediary to these stakeholders is the Canadian Residency Matching Service (CaRMS) which provides the infrastructure and the methods to mediate the career aspirations of applicants with the needs and preferences of PG programs.

But, looking closer and deeper, our national system is also impacted by the local needs and cultures of PG programs within the 17 faculties of medicine with unique circumstances and processes for selection. This only makes sense – what a family medicine program in Manitoba is seeking in a potential trainee maybe quite different from what a vascular surgery program in Montreal requires. Within clinical disciplines or within a faculty of medicine, selection processes can differ greatly depending on resourcing, culture, and context. While there have been recent attempts to inform these processes using evidence,^6^^,^^7^ we are still a long way from clarity on how best to select residents. And we are equally challenged in offering evidenced informed career counselling to UG students. Despite the volume of the literature – and debates - on postgraduate selection, the processes at both the macro and micro level are still largely a black box.

Education scholarship and research can play an important role in bringing evidence and data to these debates. The CMEJ special issue is an opportunity to open the black box and expand our understanding of the PG selection issues in Canada. All of the papers in this special issue collectively address macro or micro level issues that have previously been inside the black box known as the “match”. And notably, the papers shed a light on the issues of concern that are unique and common to all stakeholders using a variety of approaches and methods.

We received several personal and moving reflections on the match process. Silverberg in her reflection “Should I stay or Should I go” tackles a subject that is often missed and under appreciated: what happens when a resident wants to make a career change after they’ve entered training?^8^ In “‘We regret to inform you that you did not match’: reflections on how to improve the match experience,” Fellows and team analyzed on the pre- and post-match period. After going unmatched, the authors discussed the issues they faced, and offered suggestions that would have improved their experience.^9^

The papers also cover new trends that should be concern for those working to improve the selection system. In “Canadians studying medicine abroad and their journey to secure postgraduate training in Canada or the United States”, Ilona Bartman and team track the percentage of Canadians studying abroad (CSAs) that are successful in securing residency training in either Canada or the U.S. in order to provide guiding information for Canadians who are considering studying abroad.^10^ This addresses a growing concern as more and more Canadians study abroad and subsequently enter the Canadian PG selection match each year.

For medical students concerned about the match, a few papers used innovative methods to identify new insights on the match. In “Fundamental trends within falling match rates: insights from the past decade of Canadian residency matching data,” Zeng and team identified clusters of disciplines with trends in match and electives behaviours using machine learning models.^11^ They found that not all disciplines are affected equally by the declining match ratios. They hope that the results from their study will be useful in the future for reducing the number of unmatched CMGs.

“Analysis of factors affecting Canadian medical students’ success in the residency match” by Lakoff and team analyzed match outcomes of medical students to see what factors influenced an applicant’s chance of matching with their first choice discipline. They believe the results of their analysis will help guide medical students with their career planning and strategies.^12^

Medical schools may be interested in Bakker and colleagues’ study: “The relationship between regional medical campus enrollment and rates of matching to family medicine residency” which points to contextual differences between regional and main medical school campuses and then subsequently affect PG training choices.^13^

We are also very pleased to see Gallinger and colleagues report: ““CaRMS at 50: Making the match for medical education” which examines the evolution of the application and matching system over the past half century, and CaRMS' role in the process. They concluded that the system needs to evolve with future needs without compromising its current advantages.^14^

These, and the other excellent articles in this issue will hopefully provide new insights, evidence, and approaches to discuss how we can continue to improve the career development of future physicians and hence healthcare in Canada generally.

On a final note, our thanks to Drs. Jennifer O’Brien, Heather Hickey, the peer-reviewers, and authors who have helped make this issue possible.
